# A Kitchen Committee

**Published:** 1917-06-09

**Authors:** 


### FOOD IN WARTIME.*

### A Kitchen Committee.

THE success of the efforts made by the Metropolitan Asylums Board and by the East Lancashire Foodstuffs Advisory Board to improve the dietary in the institutions under their control has roused a great deal of interest. Would it not be possible, it is argued, to bring about the same good results on a smaller scale? May not an attempt be made within the walls of isolated institutions to compass the ends which have been achieved by co-operation in many? The achievements of the two bodies mentioned above are due to the principle of consultation over the meals, extended to prices, quantities required, and methods of cooking. Here and there in the institution world it is possible to meet with a matron who combines in her own person all the necessary talents for feeding her large household frugally and well. In these rare instances a benevolent despotism works admirably. But taking into consideration the enormous strain of diverse kinds which is borne by the superintendents of training-schools, it is hardly reasonable that the whole weight of directing the commissariat department should rest on their shoulders. On whose, then, should it be placed? When the steward or secretary orders the supplies, the matron orders the meals, the housekeeper gives out the stores, the cook prepares the food, the home sister does the carving, and the pigs get most of what is left, the lack of co-ordination is evident. In such households it is not considered necessary to ascertain the exact weekly average cost per head of the food. The cost is cut down to the lowest level, and if the word goes forth for increased economy, less is ordered and everyone begins to grumble. Unfortunately, however, the grumblers have no outlet. For a probationer to complain to the matron that she does not get enough to eat is unthinkable. Few sisters would like to venture on criticising the meals prepared on the order of their superior officer. These things are not done in institutions, and perhaps it would be intolerable if they were. So the grumbling goes on underhand, and things which are obviously bad and remediable never get remedied.

One way out of the difficulty appears to lie in setting up a kitchen committee charged with the duty of controlling the dietary of the staff. Its functions are (*a*) to draw up a menu stretching over a week or preferably ten days, to remain in force one or two months according to what is found most convenient; (*b*) to receive reports about prices and possible supplies from the buyer of the goods, so that the menu may be built up on economical lines; (*c*) to consult over improved methods of preparing the food, the introduction of labour-saving appliances in the kitchen, and the issuing of recipes with the necessary formulæ for preparing dishes for the numbers which have to be provided for.

It is clear that the very existence of such a kitchen committee meeting at least once a week would focus attention on numerous neglected problems which centre round the food supply. The composition of the committee, which should not be too large, is a matter varying with the size and resources of the institution. The matron, of course, presides. The steward or official charged with the duty of buying supplies should certainly be invited to attend on any occasion when the question of substituting one article of diet for another should arise, or information about cost is required. The housekeeper would be an *ex-officio* member, and the cook could learn much and teach much if, as begins now to happen in institutions, she were a woman of some education and knowledge of her art. So far, good. But to carry the staff with it the kitchen committee ought to number at least one sister and one nurse, say in her second or third year, preferably elected by their compeers, who could represent their own order and help to do some of the work which would fall on any committee which should tackle the commissariat intelligently. The value of belonging to the committee would speedily be grasped by those who thus rendered services, for it would mean an insight into rational housekeeping likely to be of use to members during their whole life.

An experiment on somewhat wider lines than the above was tried at the 5th Northern General Hospital soon after it was constituted. A separate kitchen was provided for the nurses, and a mess fund was created to which every member of the nursing staff contributed. The mess fund was managed by a sister appointed by the other members of the mess, and she was responsible for the menu and for supervising the cooking and service. We believe, however, that although found exceedingly satisfactory, the system has now been dropped. At the Lake Hospital, Ashton-under-Lyne, the plan of a food committee is now being tried, with the best results. It consists of the matron, the cook, a sister, and three probationers in their first, second, and third years. Many economies have been effected since it was instituted, war rationing has been imposed on the staff without any hardship or reluctance being experienced, and the commissariat has risen to a higher level than before in the matter of good cooking and variety.

During the present scarcity of many accustomed articles of diet, it is beyond all else expedient to make changes by consent. Where the rationing is voluntary it is carried out with a zeal which sweeps aside all notion of hardship. In institutions the only way to introduce the voluntary principle is by taking the staff into confidence and inviting them to share the labours of housekeeping under difficulties. To those who would argue that this is not the time for innovations in housekeeping, we would reply that to wait till the war is over and things become easier all round is to miss a great opportunity. There can never be a time when economy is more necessary and when new methods are more imperatively called for. And by drawing together a group of people naturally interested in their own food supplies, and inviting them to produce the best results they can for a given sum of money, economy is produced without friction.

Exact knowledge of the cost of the menu is essential to any experiments. It has been proved over and over again that a separate kitchen for the staff, though desirable in the interests of good organisation, is not a *sine qua non* in keeping accounts entirely distinct. At St. George's Hospital, for instance, where the exact cost of patients' and staff food is worked out, there is but one kitchen, and the same is true of the Royal Free Hospital. The cook directing the whole has kitchenmaids for the different tables, and when once a good system has been inaugurated, finds no difficulty in keeping all supplies distinct. The most interesting plan for a kitchen committee to work under is that of assigning a given sum per head per week within which the committee must keep, any balance which results from improved management being used for the little luxuries which help to introduce variety into the meals.

* Previous articles appeared February 24, March 10, 24, and 31, April 14 and 21, May 5, 12, and 19.

